# Physics scientific events in Brazil: Female participation

**DOI:** 10.1371/journal.pone.0287931

**Published:** 2023-07-07

**Authors:** Celia Anteneodo, Carolina Brito, Débora P. Menezes

**Affiliations:** 1 Departament of Physics, PUC-Rio, Rio de Janeiro, RJ, Brazil; 2 Institute of Physics, UFRGS, Porto Alegre, RS, Brazil; 3 Departament of Physics - CFM - UFSC, Florianópolis, SC, Brazil; UFSCar CCBS: Universidade Federal de Sao Carlos Centro de Ciencias Biologicas e da Saude, BRAZIL

## Abstract

It is known that diversity matters to improve scientific excellence and that scientific events are important occasions to discuss new ideas and create networks, beyond the fact that it helps to put the work of the scientists in evidence. Hence, increasing diversity in scientific events is crucial to improve their scientific quality and help to promote minorities. In Brazil, important physics scientific events are organized by the Brazilian Physical Society (SBF, in Portuguese), and in this work, some aspects related to the participation of women in these physics events are analyzed from 2005 to 2021. The analysis shows that women’s participation has increased over the years, reaching in some areas of Physics the same percentage as the one observed in the SBF community (always below 25%). However, female participation as members of organizing committees and as keynote speakers is always lower. Some proposals are listed to change the current picture of inequality.

## Introduction

A large number of works (see, for instance, [1–4]) reveal how important diversity is in science, both for making it better and more inclusive. Diversity allows new and different points of view and innovative research questions, thus enriching scientific development.

However, gender gaps still nowadays exist and are more prevalent in the fields of Science, Technology, Engineering, and Mathematics (STEM), according to the 2022 Globar Gender Gap Report [5]. Although the gender gap is established in several countries [6], Brazil in particular has been documented as an exceptionally unequal and hostile country for various marginalized groups, including women, LGBTQI+ individuals, Afro-descendants, indigenous communities, and people with disabilities [7–9]. Unfortunately, the Brazilian scientific community reflects this structural problem, as it remains predominantly dominated by white people [10] and male scientists [11–13]. This dominance results in underrepresentation of other groups, particularly in leadership and prestigious positions, compared to the demographic composition of the overall population [13]. To tackle the situation of women’s under-representation in these male-dominated fields, many initiatives around the world have been developed, as the *Women in STEM* in the United States and [14] and *STEM Women* in Australia [15], among many others, however, underrepresentation remains striking, therefore the continuity of these initiatives as well as the implementation of new ones are still necessary.

Particularly, with regard to scientific events, where many new ideas are inspired, diversity of race, ethnicity, religion, sex, gender, age, geographic origin, etc. is a fundamental question. In addition to this main reason, there are other reasons to be concerned about this matter. On one hand, a visible lack of diversity sends the message to potential participants that certain groups do not belong to the community, thus naturalizing partial exclusion and discouraging the participation of these sectors, which reinforces the problem. On the other hand, considering that giving good talks increases academic recognition and is important for the career promotion process, when some people are no longer invited, or no longer participate, they will have less visibility and their professional development will be penalized. Given the importance of the subject, the purpose of the present analysis is to study the level of diversity in the scientific events promoted by the Brazilian Physical Society (SBF). All events until 2019 were held in person, in 2020 and 2021 they were online. This is an aspect to be taken in mind when analyzing the data, since it has been reported that gender gaps are smaller in online than in traditional education [5], and a similar effect may occur in other activities such as in scientific events.

## Materials and methods

The analysis is based on the data stored in the SBF records obtained from the registration forms filled out to participate in SBF events within the period 2003–2022. On these forms, it currently appears as one of the questions and its response options: “Gender: Male, Female, Other”. Only on two occasions was the “Other” option registered, and this did not appear before 2021. In addition, “Sex” was asked instead of “Gender”. Therefore, the analysis will be restricted to the “Male” and “Female” categories, without any other distinction by sex or gender, not having access to data on other markers of gender diversity [16].

As a reference of the proportion of genders, the percentage of members of the SBF will be used, in each year of the studied period. Female participation in the various activities of the events (participant in any of the activities, member of committees, and speakers) will be accounted for over the years, in several areas of Physics. In the last section, actions that can be taken, at individual, collective, and institutional levels, to change this scenario of inequality will be discussed.

## Results

### SBF scientific events

A complete list of the various scientific events that are part of the official SBF calendar can be found on the SBF website [17]. In Table 1, the list of these events is shown, and it includes thematic meetings, a single regional meeting (namely, EFNNE) and thematic schools (EVJAS-FNT, EVJAS-FNE, EVJAS-PC, EBEE) mainly destined to graduate students. For each event, the table presents the annual average number, taking into account all participants over the period available in each case, and the percentage of female participants in the same period. In some events, the number of participants varied considerably during the period studied, and therefore, the averages should only be seen as indicative of the size of each community that participates in the events.

**Table 1:** Events of the official SBF calendar. The average number of participants per year in each event, and average percentage of female participation in each event are shown. Averages were calculated within the period indicated in the last column.

Name of the event in portuguese	Name of the event translated to english	Acronym	Participants anual average	Female percent	Period
Encontro Nacional de Física da Matéria Condensada / Encontro de Outono	Brazilian Meeting on Condensed Matter Physics / Autumn Meeting	ENFMC EOSBF	958	24	2005–2021
Simpósio Nacional de Ensino de Física	National Symposium on Physics Teaching	SNEF	1150	38	2005–2021
Encontro Nacional de Física de Partículas e Campos	National Meeting on Particle Physics and Fields	ENFPC	232	18	2005–2021
Reunião de trabalho sobre Física Nuclear no Brasil	Brazilian Workshop on Nuclear Physics	RTFNB	129	23	2005–2021
Encontro de Física do Norte e Nordeste	Meeting on Physics of North and Northest	EFNNE	560	25	2005–2021
Encontro de Física do Norte e Nordeste—Ensino	Meeting on Physics Teaching of North and Northest	EFNNE (Ensino)	447	35	2010–2015
Encontro de Pesquisa em Ensino de Física	Meeting on Physics Teaching Research	EPEF	267	41	2016–2020
Escola de Verão Jorge André Swieca Física Nuclear Teórica	Jorge André Swieca Summer School on Theoretical Nuclear Physics	EVJAS-FNT	58	13	2015–2021
Escola de Verão Jorge André Swieca Física Nuclear Experimental	Jorge André Swieca Summer School on Experimental Nuclear Physics	EVJAS-FNE	24	29	2016–2020
Escola de Verão Jorge André Swieca Partículas e Campos	Jorge André Swieca Summer School on Particles and Fields	EVJAS-PC	91	19	2015–2021
Escola Brasileira de Estrutura Eletrônica	Brazilian School on Electronic Structure	EBEE	120	36	2016–2021

**Fig 1:**
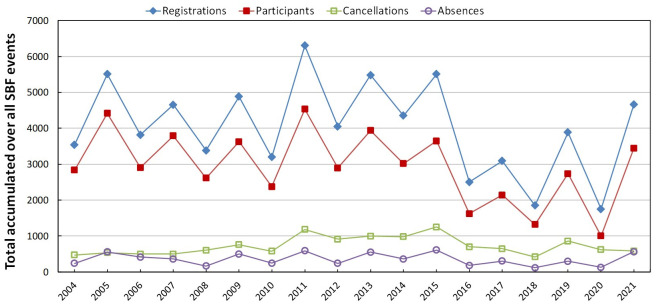
Numbers of participants in SBF events. Total of participants per year, accumulated over all SBF events, in each year, within the period 2004–2020. The total numbers of registrations, participants, cancellations, and absences are displayed.

Fig 1 depicts the temporal evolution of the total number of participants in the various SBF events, grouping the data by year. The oscillations are due to the fact that some events, such as the SNEF and the Jorge André Swieca (EVJAS) Summer Schools, are biennial, and thematic events, such as the EFNNEs are not regularly held every year.

The effective number of participants corresponds to those initially enrolled minus cancellations and absences, numbers also shown Fig 1. Despite the large fluctuations, there has been a clear downward trend in participation in these national events in recent years. Sporadically (2011 and 2016), it has been held the National Physics Meeting (familiarly called “Encontrão”), which brings together all the areas of Physics (all thematic events). However, each thematic event within the Encontrão keeps its own records, allowing a separate analysis of each one of them.

**Fig 2:**
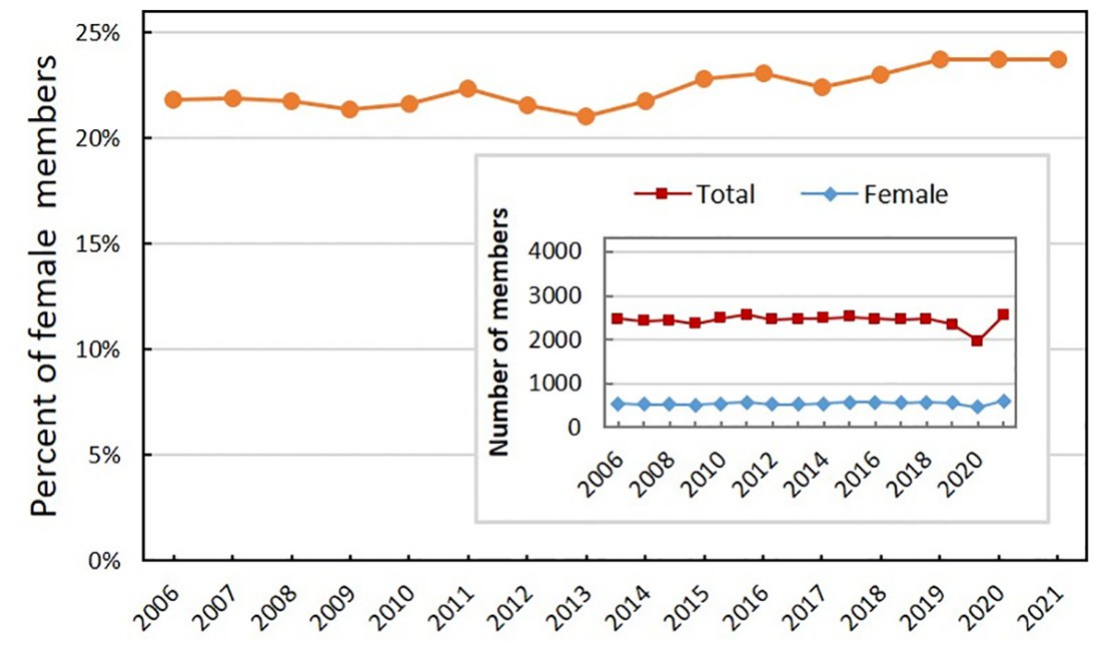
Composition of SBF members. Percentage of SBF members, in the period 2003–2022. The inserted graph shows the total number of non-defaulting members (including both sexes) and the number of non-defaulting female members.

As a reference, to estimate the composition by gender of the SBF community, Fig 2 presents the percentage of female members over the period 2006–2021. The inserted graph shows the total number of SBF members (non-defaulting members of both genders considered) and the total number of non-defaulting female members. These numbers remain nearly stable over the years, except for fluctuations during the pandemic in 2020–2021. According to linear regressions (not shown), there is a small downward trend in the total number of members in recent years (except for circumstantial peaks, generally related to the holding of large physics meetings), while in the subgroup of female members, there is a very subtle increase. Therefore, the female proportion shows a slight increase in recent years but remained stable during the pandemic. However, this increase may be due to a change in the composition of the community of members, with the incorporation of colleagues from the teaching area. Notice that, in this community, as can be seen in Table 1, female participation is greater than in the entire SBF community, with 38% in the SNEF (largest national event in the area of education in Physics) and 41% in EPEF (on research in this area), both being biennial, and 35% in ENFNNE-Ensino, carried out between 2010 and 2015.

In most thematic meetings and in the regional one (EFNNE), the female proportion is in the range of 23 to 25% (see Table 1), consistent with the percentage of female members shown in Fig 2, except in the case of the ENFPC, with 18% of female participants, slightly below the percentage of members.

In the schools, the proportion of female participants presents a wider variation, between 13 and 36%. It should be noted that the size of the schools is typically smaller than that of the meetings, and the community of participants is mostly made up of students, in varying numbers.

### Composition per activity developed in the event

For the various events, in addition to the proportion of women among the participants (in all forms of participation), the proportions in the committees and in the list of invited speakers have been considered. The set of committee members includes all types of participation in committees: coordinators (general and program), area, regional and other committees, and the structure of committees can change from one event to another and from one edition to another of the same event. Invited talks include plenary and parallel oral presentations. The other types of oral presentations and posters were not contabilized.

**Fig 3:**
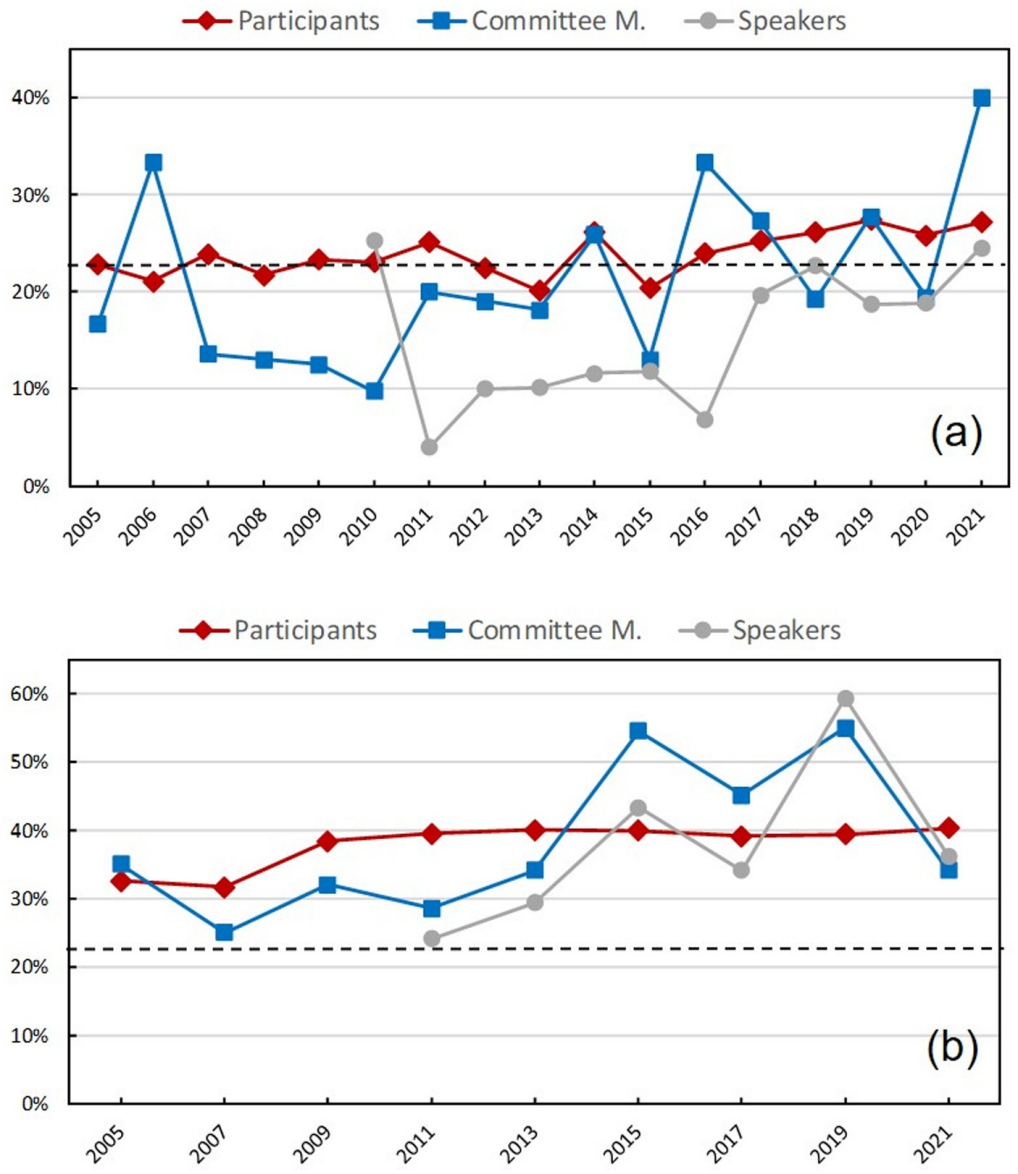
Percentage of females in the largest events. (a) general physics (ENFMC-EOSBF) and (b) physics teaching (SNEF). Information about speakers prior to 2010 is lacking. The percentage of total participants (red diamonds), members of committees (blue squares), and speakers (light gray circles) are plotted joined by lines as guide to the eye. The average percentage of female members of SBF in 2005–2021 (22.4%) is plotted for comparison (dashed line).

Fig 3(a) presents the results for the ENFMC-EOSBF. The National Meeting of Condensed Matter Physics (ENFMC), which since 2017 has come to encompass other areas and has been called the SBF Autumn Meeting (EOSBF) [18], is one of the most traditional SBF events. It currently comprises the following areas: atomic and molecular physics, biological, statistical and computational, condensed matter and materials, medical physics, physics in enterprises, optics and photonics. The plasma physics community also participates in the EOSBF, every two years. This figure shows the female percentages in the different sets of participants of the ENFMC-EOSBF (all participants, committee members and speakers). We observe in the figure that, in the ENFMC-EOSBF, the female percentage of participants (red symbols) reflects the representativeness of the group among the SBF members. In fact, over the years, the red curve follows closely the average value given by the dashed line as well as the subtle increase of female SBF members in recent years, observed in Fig 1. With regard to female participation in committees (blue symbols), observe that it has been noticeably lower than the total participation (red symbols), over all the years up to approximately 2013, except for the high peak in 2006. After 2013, female participation in committees fluctuates around the percentage of overall participants. Female speakers (light gray symbols) are neatly even more underrepresented in percentage lower than that of participants over all years except in 2010. In each year of the period 2011–2016, the percentage of speakers is approximately half that of participants. After 2016, there is an improvement, with the percentage of speakers approaching that of the female participants, but still remaining below.

For the SNEF, a meeting from the teaching area, the same previous analysis is presented in Fig 3(b). As previously seen regarding Table 1, in the area of the SNEF, female participation is greater than in other areas, having increased and remained close to 40% in recent years. However, until 2013 (except in 2005), the proportion of female participants in committees is lower than the proportion of female participants, and the proportion of speakers even lower than that in committees (compare the curves, one above the other, for the period). Already after 2013, in each year, the numbers of female organizers and speakers fluctuate slightly above the (red) level that represents the percentage of female participants. Let us remark that, in 2021, despite the online format, the fractions of female organizers and speakers have both fallen below that of participants.

The results for other events (EFNNE, ENFPC and RTFNB) are shown in S1 Fig. The size of these events is smaller than those of EOSBF and SNEF, hence fluctuations are larger, but manifest similar trends. In the case of the regional event EFNNE (north and northest), shown in S1(a) Fig, we clearly see that the curve of female participants is above those of committee members and speakers, for all years except in 2021, when the relation is inverted. Particularly, except in a few editions, the proportion of speakers over the years is the lowest, even null. The exception and apparent improvement observed in 2021, however, may be a consequence of the online format, which in some way encourages participation that otherwise may encounter obstacles. For the ENFPC (particles and fields), in S1(b) Fig, note that except in 2006 and 2014, the participation in committees is lower than that of participants, which in all years is below the average in the whole SBF community (red symbols below dashed line). Female speakers are predominantly underrepresented over the year with a neat improvement after 2017. For the RTFNB (nuclear physics), in S1(c) Fig, the female participation in committees presents positive and negative differences with respect to the female participation in the event, however, we highlight that the participation as speakers is systematically below the previous numbers, with improvement after 2019 when the proportion of female speakers nearly coincides with the proportion of female participants.

## Discussion and final remarks

In this work, female participation in SBF scientific events, which are among the largest and most prestigious national events in Brazil has been quantified. An imbalance, especially in event management activities and keynote speakers is clearly noted. Female participation in event committees is lower than male participation. In recent years, however, the proportion has approached that of SBF affiliates. Nevertheless, the proportion of female speakers, despite having increased in recent years (as can be observed in almost all the figures), remains low in comparison with the female percentage of participants. These observations are more pronounced outside the teaching area where the female group is better represented.

These findings are neither exclusive to the field of physics, nor an isolated case observed only in Brazil. The lack of gender diversity in scientific events has been identified in previous works [19–24] and is a problem for several reasons. One of them is that giving lectures and especially being an invited speaker are academic recognitions and are relevant in career progression. Having less opportunity to expose one’s own work is, therefore, one of the mechanisms that lead to the reduction of women throughout their careers, known as the Scissors Effect [12, 25, 26] and contributes to the crystal ceiling. Another reason, not less relevant, is that a smaller range of points of view receive visibility. Therefore, the present analysis is important to make the physics community aware of the problem and seek to take actions intending to correct the low female participation, and other gender disparities in events.

Several studies identify a correlation between the proportion of women among event organizers and the proportion of women among guest speakers [19–21, 24]. In particular, in [24] it is shown that the presence of at least one woman in the organization team is correlated with a significantly higher proportion of guest speakers and a lower probability of having events with exclusively male guests. These findings point to the need for a more diverse composition, in all aspects, of the organization of scientific events.

The above suggestion might lead you to think that this is a problem that can only be solved by women, but this is not the case. It is crucial that the problem is recognized throughout the community and that everyone works together to make it more inclusive and diverse. Some attitudes at the individual level can contribute to changing the scenario. Each interested and potential event participant can stay tuned, evaluate the level of diversity, and if necessary, contact the organizers to suggest new names for speakers and committee members. This attitude, in addition to contributing directly to increasing diversity and inclusion, conveys the message that this aspect is considered relevant by the community.

It is worth noting that part of the efforts to increase female participation may be limited by the fact that female guests tend to take longer to respond and reject invitations more often than males, as noted by some scientists in the physics community who participate in the organization of events, but there are no available statistics about it. One hypothesis to explain this observation is that there are proportionally few women who reach the highest levels in their careers and the resulting professional recognition [26]. Thus, these few women are often overburdened, while other younger or less well-known female researchers are not encouraged. One way to avoid this problem is to build a list of women to be invited and ask people in the community to contribute to setting up a database. Including young researchers with great potential and excellent contributions makes it possible to give space to more women and share the burden with those who no longer have space on the agenda.

Institutional actions can also be carried out. Scientific societies should prepare (those that do not yet have) a guide to equal opportunity guidelines, similar to what exists in some societies such as the Brazilian Physical Society [27, 28] and the American Physical Society [29]. Once this guide has been prepared, containing suggestions on how to organize more diverse events, and other information, it must be constantly discussed and disseminated to make clear the appreciation of equal opportunities in science. It is also crucial that funding agencies and scientific organizations that finance events make explicit the need for diversity in such events in their aid notices, following the example of what IUPAP (The International Union of Pure and Applied Physics) does [30]. An important initiative that may help future participation of parents of small children in events is that some Bazilian funding agencies already accept that children can be supported to accompany their parents.

Finally, it is clear that these actions to increase diversity must be applied equally to other sections of the community, besides sex and gender groups, for example, ethnic, geographic, by institution, by age, etc.

## Supporting information

S1 Fig Percentage of females in other SBF events. (a) EFNEE, (b) ENFPC, and (c) RTFNB. The percentage of total participants (red diamonds), members of committees (blue squares), and speakers (light gray circles) are plotted joined by lines as guide to the eye. The average percentage of female members of SBF in 2005-2021 (22.4%) is plotted for comparison (dashed line).
(TIF)
