# Supplementary figures and images for: Physics scientific events in Brazil: Female participation
Source: PLoS One. 2023 Jul 7;18(7):e0287931. doi: 10.1371/journal.pone.0287931 (PMC10328322; doi:10.1371/journal.pone.0287931)

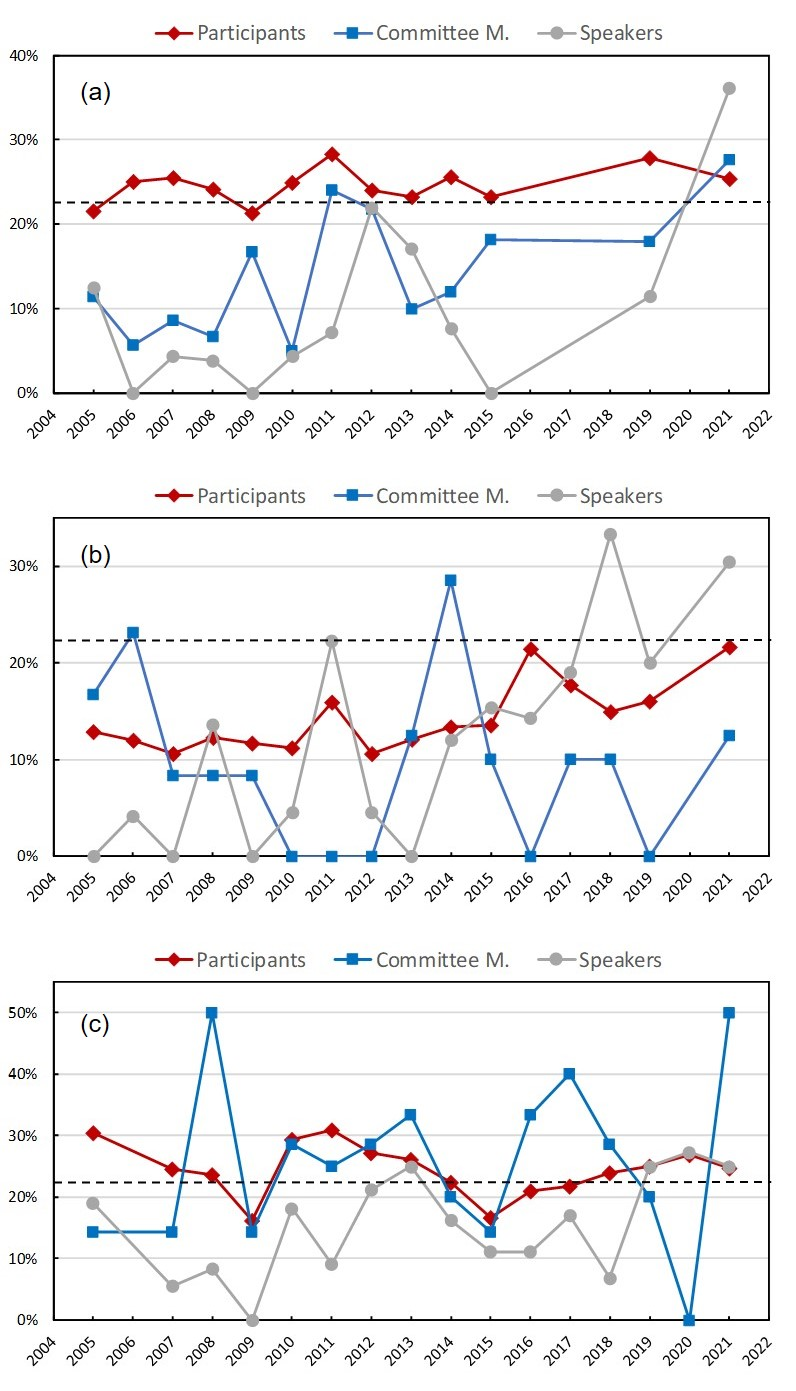

Supplement: S1 Fig — (a) EFNEE, (b) ENFPC, and (c) RTFNB. The percentage of total participants (red diamonds), members of committees (blue squares), and speakers (light gray circles) are plotted joined by lines as guide to the eye. The average percentage of female members of SBF in 2005-2021 (22.4%) is plotted for comparison (dashed line). (TIF) [file pone.0287931.s001.tif]
